# Alzheimer’s Disease Risk Polymorphisms Regulate Gene Expression in the *ZCWPW1* and the *CELF1* Loci

**DOI:** 10.1371/journal.pone.0148717

**Published:** 2016-02-26

**Authors:** Celeste M. Karch, Lubov A. Ezerskiy, Sarah Bertelsen, Alzheimer’s Disease Genetics Consortium (ADGC), Alison M. Goate

**Affiliations:** 1 Department of Psychiatry, Washington University School of Medicine, St. Louis, Missouri, United States of America; 2 Hope Center Program on Protein Aggregation and Neurodegeneration, Washington University School of Medicine, St. Louis, Missouri, United States of America; 3 Department of Neuroscience, Icahn School of Medicine at Mount Sinai, 1425 Madison Avenue, New York, NY 10029, United States of America; Central China Normal University, CHINA

## Abstract

Late onset Alzheimer’s disease (LOAD) is a genetically complex and clinically heterogeneous disease. Recent large-scale genome wide association studies (GWAS) have identified more than twenty loci that modify risk for AD. Despite the identification of these loci, little progress has been made in identifying the functional variants that explain the association with AD risk. Thus, we sought to determine whether the novel LOAD GWAS single nucleotide polymorphisms (SNPs) alter expression of LOAD GWAS genes and whether expression of these genes is altered in AD brains. The majority of LOAD GWAS SNPs occur in gene dense regions under large linkage disequilibrium (LD) blocks, making it unclear which gene(s) are modified by the SNP. Thus, we tested for brain expression quantitative trait loci (eQTLs) between LOAD GWAS SNPs and SNPs in high LD with the LOAD GWAS SNPs in all of the genes within the GWAS loci. We found a significant eQTL between rs1476679 and *PILRB* and *GATS*, which occurs within the *ZCWPW1* locus. *PILRB* and *GATS* expression levels, within the *ZCWPW1* locus, were also associated with AD status. Rs7120548 was associated with *MTCH2* expression, which occurs within the *CELF1* locus. Additionally, expression of several genes within the *CELF1* locus, including *MTCH2*, were highly correlated with one another and were associated with AD status. We further demonstrate that *PILRB*, as well as other genes within the GWAS loci, are most highly expressed in microglia. These findings together with the function of PILRB as a DAP12 receptor supports the critical role of microglia and neuroinflammation in AD risk.

## Introduction

Late onset Alzheimer’s disease (LOAD) is a complex, heterogeneous disease with a strong genetic component (reviewed in [1]). *APOEε4* is the strongest genetic risk factor for LOAD: carrying one copy of *APOEε4* increases AD risk by 3 fold and carrying two copies of *APOEε4* increases AD risk by 8–10 fold (reviewed in [2]). However, only 50% of LOAD cases carry an *APOEε4* allele, suggesting that other genetic factors contribute to risk for LOAD.

In the last six years, genome wide association studies (GWAS) have facilitated the analysis of millions of single nucleotide polymorphisms (SNPs) in tens of thousands of samples [3–10]. The International Genomics of Alzheimer’s Project (IGAP) has recently applied this approach to LOAD case and control studies in 74,046 individuals, revealing 21 loci that modify LOAD risk: *ABCA7*, *APOE*, *BIN1*, *CASS4*, *CD2AP*, *CD33*, *CELF1*, *CLU*, *CR1*, *EPHA1*, *FERMT2*, *HLA-DRB5/DRB1*, *INPP5D*, *MEF2C*, *MS4A6A*, *NME8*, *PICALM*, *PTK2B*, *SLC24A4*, *SORL1*, and *ZCWPW1* [9]. The IGAP GWAS genes fall into several common pathways that have been previously implicated in AD: neural development, synapse function, endocytosis, immune response, axonal transport, and lipid metabolism (reviewed in [1]). However, the specific effects of these SNPs on gene function and the resulting impact on disease remains poorly understood [11–14]. Two aspects of GWAS approaches have limited the interpretations that we can make regarding the functional impact of these SNPs on the molecular mechanisms underlying AD. First, the majority of the most significant GWAS SNPs are located in non-coding or gene-dense regions, making it challenging to identify which gene the SNP is modifying. Second, the majority of GWAS top SNPs are in high linkage disequilibrium (LD) with many SNPs, which in some cases span hundreds of kilobases, making it difficult to determine which SNP is the functional variant responsible for modifying LOAD risk.

Our group and others have previously demonstrated that some LOAD GWAS genes are differentially expressed in AD brains [11, 15, 16]. We found that expression levels of some LOAD GWAS genes that were identified in early GWAS [3–8], including *ABCA7*, *BIN1*, *CD33*, *CLU*, *CR1*, and *MS4A6E*, are associated with clinical and/or neuropathological aspects of AD [15] but failed to identify strong expression quantitative trait loci (eQTLs) [15, 17].

Despite the identification of additional, novel GWAS loci that modulate LOAD risk, we still know little of the functional impact of LOAD GWAS SNPs and the role of these genes in AD pathogenesis. We sought to examine functional effects of IGAP GWAS SNPs by examining eQTLs in several human brain expression cohorts. To do this, we identified all of the genes that fell within the LD block for each IGAP GWAS locus. We then analyzed eQTLs and association with AD status. rs1476679 and rs7120548 are associated with *PILRB* and *MTCH2* expression, respectively. Additionally, the expression of several genes within the *CELF1* locus, including *MTCH2*, were highly correlated and were associated with AD status. Importantly, these significant eQTLs and expression differences in LOAD brains were observed in genes that occur within the IGAP GWAS loci but not the named IGAP GWAS gene. Together, our findings demonstrate that several LOAD risk variants modify expression of nearby genes and may contribute to LOAD risk.

## Results

### Identifying genes associated with IGAP GWAS SNPs

A recent IGAP GWAS in 74,046 individuals revealed 21 loci that are significantly associated with altered AD risk, 12 of which are novel [9]: *ABCA7*, *APOE*, *BIN1*, *CASS4*, *CD2AP*, *CD33*, *CELF1*, *CLU*, *CR1*, *EPHA1*, *FERMT2*, *HLA-DRB5/DRB1*, *INPP5D*, *MEF2C*, *MS4A6A*, *NME8*, *PICALM*, *PTK2B*, *SLC24A4*, *SORL1*, and *ZCWPW1*. To define the functional impact of the IGAP SNPs, we used RegulomeDB and HaploReg to predict the regulatory potential of the IGAP SNPs (S1 Table) [18]. One IGAP SNP, rs1476679, produced a RegulomeDB score with suggestive regulatory potential (Score: 1f; Table 1)[19]. RegulomeDB predicts that rs1476679 affects protein binding of RFX3, FOS and CTCF and exhibits eQTLs with *GATS*, *PILRB*, and *TRIM4* (Table 1). Rs8093731 modifies a PAX6 motif and protein binding of E2F4 and FOS (Score: 2b; Table 1). Rs10792832 modifies an *FAC1* motif and binding of SPI1 (Score: 3a; Table 1). Despite the identification of several SNPs that have suggestive regulatory potentials, we were unable to identify eQTLs in either RegulomeDB or HaploReg that occur within the named LOAD GWAS gene (Table 1).

**Table 1:** Regulatory effects of IGAP top SNPs.

		RegulomeDB				HaploReg		
IGAP Gene	IGAP SNP	Score	eQTL	Motif Changed	Proteins Bound	eQTL	Motifs Changed	Proteins Bound
ZCWPW1	rs1476679	1f	*TRIM4*, *PILRB*, GATS*	-	CTCF, FOS, RFX3	-	-	CTCF
DSG2	rs8093731	2b	-	PAX6	E2F4, FOS	-	AHR, NKX2, NKX3, PAX6, PBX3	-
PICALM	rs10792832	3a	-	FAC1	SPI1	-	AP-3, FAC1, HDAC2	-
MS4A6A	rs983392	4	-	-	RUNX1	-	HMG-IY, HAND1, MYC	-
ABCA7	rs4147929	4	-		MAZ, IRF1	-	HNF4,SP2	-
CR1	rs6656401	5	-	-	-	-	RXRA,YY1	-
BIN1	rs6733839	5	-	MEF2, PU.1	-	-	DOBOX4, MEF2, NFκB, VDR	-
EPHA1	rs11771145	5	-	-	-	-	HOXD10	GATA2
CLU	rs9331896	5	-	-	-	-	BDP1, NRSF	-
CD33	rs3865444	5	-	-	-	-	CDP, FOXO, SREBP	-
HLA	rs9271192	5	-		CHD1, MXI1, TBP	-	HOXA13, POU2F2, TCF11::MAFG	POL2
PTK2B	rs28834970	5	-	-	-	-	CEBPA, CEBPB, CEBPD, HSF,STAT,P300	-
SORL1	rs11218343	5	-	-	POLR2A, TBP, RFX3	-	-	-
SLC24A4/RIN3	rs10498633	5	-	-	-	-	AP1, CDX2, FOXD1, FOXJ2, HOXA9, HOXC10, HOXC9, MRG1:HOXA9, NKX6, PDX1, TCF12, P300	-
INPP5D	rs35349669	5	-	RBP-Jκ	-	-	AP-2rep,RBP-Jκ	-
FERMT2	rs17125944	5	-	-	-	-	PU.1, SRF, P300	-
CASS4	rs7274581	5	-	-	-	-	E2F, SIN3AK-20, YY1	-
CD2AP	rs10948363	6	-	FOXJ3, TCF3	-	-	FOXJ1, HOXB13, SOX	-
CELF1	rs10838725	6	-	C/EBPΔ, FOXA2, HNF3β	-	-	CEBPB, CEBPD, Foxa	-
MEF2C	rs190982	7	-	-	-	-	GATA, HNF1	-
NME8	rs2718058	7	-	-	-	-	AP1, ELF3, FOXA, HMG-IY, MEF2, PAX6, STAT	-

*Monocytes. PU.1 is the protein product of SPI1

The majority of GWAS SNPs occur in regions of high LD that span multiple genes [9]. Thus, we asked whether IGAP GWAS SNPs alter expression of genes that are within the LD block rather than the genes immediately under the SNP with the highest p-value. Manhattan plots reported in Lambert et al. were used to identify all of the genes within the LD block for each IGAP GWAS SNP (Table 2) [9]. Eleven of the 21 IGAP GWAS SNPs have multiple genes within the LD block. We tested whether the IGAP GWAS SNPs have functional effects on gene expression by examining all of the genes within each region.

**Table 2:** Genes within the IGAP GWAS loci.

IGAP SNP	IGAP Gene	Genes within LD block
rs6656401	*CR1*	*CR2*, *CR1L*
rs6733839	*BIN1*	*CYP27C1*
rs10948363	*CD2AP*	*None*
rs11771145	*EPHA1*	*LOC285965*, *TAS2R60*
rs9331896	*CLU*	None
rs983392	*MS4A6A*	*MS4A3*, *MS4A2*, *MS4A6A*, *MS4A4A*, *MS4A6E*
rs10792832	*PICALM*	*EED*
rs4147929	*ABCA7*	*CNN2*, *POLR2E*, *GPX4*, *HMHA1*, *SBNO2*
rs3865444	*CD33*	None
rs9271192	*HLA-DRB5–HLA-DRB1*	*HLA-DRB6*, *HLA-DQA1*, *HLA-DQB1*
rs28834970	*PTK2B*	None
rs11218343	*SORL1*	None
rs10498633	*SLC24A4 & RIN3*	None
rs8093731	*DSG2*	*DSG3*
rs35349669	*INPP5D*	None
rs190982	*MEF2C*	None
rs2718058	*NME8*	*GPR141*
rs1476679	*ZCWPW1*	*NYAP1*, *PMS2P1*, *PILRB*, *PILRA*, *C7ORF61*, *C7ORF47*, *MEPCE*, *GATS*
rs10838725	*CELF1*	*MADD*, *SLC39A13*, *PSMC3*, *NDUFS3*, *KBTBD4*, *PTPMT1*, *MTCH2*, *AGBL2*, *FNBP4*, *NUP160*, *C1QTNF4*, *RAPSN*
rs17125944	*FERMT2*	None
rs7274581	*CASS4*	*C20ORF43*, *CSTF1*

### eQTLs in AD Risk Loci

To determine whether IGAP GWAS SNPs modify expression of genes within the GWAS loci, we examined cis-eQTLs in a publically available dataset from neuropathologically confirmed normal control brains (UKBEC [20]; Table 3 and S2 Table). Rs1476679 was significantly associated with expression of multiple *PILRB* transcripts in most brain regions (Table 3). Several transcripts shared between *PILRB* and *PILRA* were associated with rs1476679. However, transcripts specific to *PILRA* did not exhibit an eQTL with rs1476679, suggesting that the effect is driven by differences in *PILRB* specifically (Table 3). *GATS*, which is also present within the LD block of the IGAP SNP, had a single transcript that also displayed an eQTL with rs1476679 in most brain regions (Table 3). The IGAP SNP, rs9331896, was significantly associated with *CLU* expression in the white matter, hippocampus, temporal cortex and occipital cortex (Table 3). EQTLs were also observed between rs6656401 and *CR1*, *CR2* and *CR1L*, all of which occur within the LD block for rs6656401. The IGAP SNP rs10838725, occurs in a gene dense region and several genes within this region exhibited eQTLs with the IGAP SNP: *CELF1*, *NDUF3*, *KBTD4*, *PTPMT1*, *MTCH2*, *FNBP4*, *MADD* and *NUP160* (Table 3). IGAP SNPs rs983392, rs10792832, rs2718058, and rs7274581 exhibited eQTLs with *MS4A6A*, *EED*, *GPR141*, and *CASS4*, respectively (Table 3). Although several loci showed nominal association, only the *CR1* eQTL in WHMT survived a strict multiple test correction (Bonferroni p = 3.9x10^-5^).

**Table 3:** eQTLs of IGAP GWAS SNPs in control brains (UKBEC).

				Brain Region (P value)									
IGAP SNP	Gene	Transcript	Probe ID	FCTX	TCTX	HIPP	PUTM	THAL	MEDU	SNIG	WHMT	CRBL	OCTX
rs6656401	CR1*	t2377332	2377395	**0.03**	0.11	**0.01**	0.13	0.88	0.53	0.91	**3.70x10**^**-7**^	0.58	0.21
			t2377332	**0.01**	**4.00x10**^**-3**^	**2.70x10**^**-5**^	**0.05**	**0.01**	0.09	0.15	**6.50x10**^**-7**^	0.31	0.07
	*CR2*	t2377283	2377285	0.69	0.39	0.73	0.77	0.68	0.69	**3.80x10**^**-3**^	0.63	0.11	0.11
	*CR1L*	t2377527	2377428	0.36	0.97	0.27	0.42	**6.80x10**^**-3**^	0.13	0.80	0.54	0.70	0.66
		t2377427	2377445	0.31	0.09	0.93	0.72	0.47	0.71	0.14	**6.30x10**^**-4**^	0.38	0.28
rs9331896	CLU*	t3129065	3129079	**0.04**	**1.30x10**^**-3**^	**1.50x10**^**-3**^	0.27	0.69	0.50	0.14	**3.50x10**^**-4**^	0.89	0.07
			t3129065	**1.20x10**^**-3**^	**4.50x10**^**-4**^	**6.90x10**^**-4**^	0.94	0.14	0.62	**0.03**	**1.50x10**^**-4**^	**0.08**	**7.70x10**^**-4**^
rs10792832	PICALM*	No eQTL											
	*EED*	t3343202	t3343202	0.98	0.22	0.56	**4.10x10**^**-3**^	**4.40x10**^**-3**^	**2.10x10**^**-4**^	0.12	0.12	0.44	0.36
rs4147929	ABCA7*	No eQTL											
	*CNN2*	No eQTL											
	*GPX4*	No eQTL											
	*HMHA1*	No eQTL											
	*POLR2E*	t3844952	t3844952	0.06	**3.7x10**^**-3**^	0.71	0.06	**1.80x10**^**-3**^	0.52	0.71	0.22	0.91	0.52
			3844957	0.61	**0.05**	0.02	0.65	0.32	1.00	0.57	**1.1x10**^**-3**^	0.79	0.93
			3844969	0.91	0.26	0.91	**8.80x10**^**-3**^	0.91	0.67	0.30	0.18	0.44	**0.02**
	*SBNO2*	t3844978	t3844978	0.74	0.80	0.30	0.70	0.76	0.23	**5.0x10**^**-3**^	0.68	0.89	0.42
rs2718058	NME8*	No eQTL											
	*GPR141*	t2997789	2997791	**0.02**	0.11	0.35	**8.50x10**^**-3**^	0.95	0.33	0.23	0.67	0.64	0.39
		t2997811	2997812	0.80	0.51	**0.05**	0.29	0.30	0.98	**2.30x10**^**-3**^	**7.50x10**^**-3**^	0.43	0.65
rs1476679	ZCWPW1*	t3063968	3063971	**1.10x10**^**-3**^	0.30	0.84	0.33	0.06	0.36	0.77	0.06	0.09	0.70
			t3063968	0.06	0.66	0.90	0.29	0.33	0.82	**1.30x10**^**-3**^	0.70	0.83	0.67
	*PILRB/PILRA*	t3015519	3015527	**2.30x10**^**-3**^	**0.02**	**3.10x10**^**-3**^	**1.10x10**^**-3**^	**6.40x10**^**-4**^	0.1	**9.90x10**^**-3**^	0.12	**5.80x10**^**-4**^	**0.02**
			3015536	**8.30x10**^**-4**^	**0.04**	0.16	0.39	0.12	0.5	0.05	0.5	0.91	0.32
	*PILRB*	t3015442	3015442	0.13	0.094	**3.1x10**^**-3**^	**0.043**	**0.045**	0.36	**0.023**	0.16	**0.03**	0.34
			3015476	0.94	0.07	0.34	0.93	0.72	0.24	0.76	0.65	0.2	0.29
			3015452	**0.04**	**1.20x10**^**-3**^	**5.90x10**^**-4**^	**4.20x10**^**-4**^	**5.6x10**^**-3**^	0.07	0.3	**0.01**	**1.60x10**^**-3**^	**9.50x10**^**-4**^
	*PILRA*	t3015543	3015543	0.9	0.59	0.23	0.94	0.68	0.28	0.78	0.74	0.78	0.24
			3015544	0.9	0.59	0.23	0.94	0.68	0.28	0.78	0.74	0.78	0.24
	*GATS*	t3063856	3063856	0.2	**0.03**	**0.04**	0.06	**9.9x10**^**-3**^	**3.5x10**^**-4**^	**0.73**	**0.04**	0.43	0.14
			3063857	**0.02**	0.55	0.72	0.58	0.91	0.52	0.16	0.56	**0.01**	0.1
			3063864	**6.5x10**^**-3**^	**0.01**	**0.05**	**0.03**	**2.0x10**^**-3**^	**2.4x10**^**-5**^	0.56	**0.02**	**0.04**	0.06
			3063864	**0.02**	**0.05**	**0.03**	**2.00x10**^**-3**^	**2.40x10**^**-5**^	0.56	**0.01**	**0.04**	0.06	**6.50x10**^**-3**^
	*MEPCE*	No eQTL											
rs10838725	CELF1*	t3372253	3372283	0.43	0.80	0.32	0.89	0.25	**2.30x10**^**-3**^	0.81	0.12	0.85	0.57
			t3372253	0.83	0.46	0.68	0.86	**1.20x10**^**-3**^	**4.00x10**^**-3**^	0.80	0.28	0.71	0.86
	*MADD*	t3329724	3329744	0.57	0.49	0.97	0.40	0.52	**5.90x10**^**-3**^	0.99	0.99	0.36	0.61
	*NDUFS3*	t3329904	3329922	0.76	0.34	0.96	0.93	0.21	**2.60x10**^**-3**^	0.88	0.11	0.15	0.27
	*KBTD4*	t3372337	3372347	0.75	0.91	0.93	0.70	0.90	0.76	1.00	0.16	**1.10x10**^**-3**^	0.82
	*PTPMT1*	t3372006	3372066	0.86	**7.90x10**^**-3**^	0.12	0.67	0.55	**0.04**	1.00	0.84	0.90	0.96
			3372037	0.86	0.14	0.74	0.67	**6.70x10**^**-3**^	**0.02**	0.78	0.10	0.40	0.19
			3372007	0.92	0.13	0.14	0.46	0.07	0.26	0.29	0.72	**2.50x10**^**-3**^	0.34
			3372006	0.31	0.77	0.68	0.93	0.31	**9.10x10**^**-4**^	0.65	0.98	0.61	0.39
	*MTCH2*	t3372368	3372370	0.78	**9.50x10**^**-3**^	0.06	0.32	**7.20x10**^**-3**^	**1.40x10**^**-3**^	0.60	0.13	0.17	0.20
	*FNBP4*	t3372459	3372495	0.79	0.67	0.51	0.41	**1.30x10**^**-3**^	**3.30x10**^**-3**^	0.66	0.16	0.40	0.10
			3372515	0.56	0.22	**5.10x10**^**-4**^	0.94	**0.03**	0.16	0.83	0.07	0.77	**2.90x10**^**-4**^
			t3372459	0.96	0.43	0.86	0.72	**3.90x10**^**-3**^	**3.60x10**^**-4**^	0.66	0.24	0.95	0.13
	*NUP160*	t3371986	3372006	0.31	0.77	0.68	0.93	0.31	**9.10x10**^**-4**^	0.65	0.98	0.61	0.39
	*SLC39A13*	No eQTL											
	*PSMC3*	No eQTL											
	*AGBL2*	No eQTL											
	*C1QTNF4*	No eQTL											
	*RAPSN*	No eQTL											

*IGAP Gene. No eQTL indicates p value was greater than 0.05 in all brain regions. P values reported for all IGAP SNPs and genes within each loci in Supplemental Table 2. Bonferroni p = 3.9x10^-5^

Our initial analyses were performed using the candidate genes manually selected from genes within the IGAP GWAS loci. We next applied an unbiased approach to determine which genes are most highly associated with the IGAP GWAS SNPs (UKBEC; S3 Table). A subset of the IGAP SNPs, rs6656401, rs9331896, rs28834970, and rs10498633, and rs190982, were associated with expression of the named IGAP gene, *CR1*, *CLU*, *PTK2B*, *SLC24A4*, and *MEF2C*, respectively (S3 Table). For the majority of the IGAP SNPs, genes that occur within the LD block for the IGAP GWAS loci are among the ten most highly associated eQTLs; however, the associations failed to achieve statistical significance (S3 Table).

In order to replicate these eQTL findings, we analyzed a second publically available dataset composed of expression and genotype information from neuropathologically normal control brains (GSE15745 [21]). In most cases, the original GWAS SNP was not present in the dataset; so, we used one or more SNPs in high LD with the GWAS SNP to test for eQTLs (Table 4; S4 Table). Cis-eQTLs were analyzed in frontal and temporal cortices (Table 4; S4 Table). We observed a significant association between rs5015756, in LD with IGAP SNP rs1476679 (r^2^ = 0.8; D’ = 1; S5 Table), and several *PILRB* probes (Table 3; p = 3.26x10^-5^, FCTX, and 4.12x10^-5^, TCTX; Bonferroni p = 3.2x10^-4^).

**Table 4:** SNPs in LD with rs1476679 produce eQTL with PILRB in control brains (GSE15745).

Analyzed SNP	PILRB Transcript	Frontal Cortex		Temporal Cortex	
		P value	β	P value	β
rs5015756	ILMN_1768754	0.2952	0.0375	**0.0182**	**0.1035**
	ILMN_1685534	**5.65x10**^-5^*	**0.0678**	0.0572	0.0274
	ILMN_1723984	**3.26x10**^-5^*	**0.0811**	**4.12x10**^-5^*	**0.0575**
	ILMN_1760345	**0.0384**	**0.0462**	0.4359	0.0101
	ILMN_1729915	0.7101	-0.0046	0.5242	0.0084
	ILMN_1663753	0.0732	0.0213	**0.0257**	**0.0309**

* Passed multiple test correction (Bonferroni p = 3.2x10^-4^)

In a third replication dataset containing expression and genotype information from AD and control brains (GSE15222), we were able to replicate the eQTL between rs1476679 and *PILRB* (p = 0.0022; Table 5; Bonferroni p = 0.003). Additionally, we replicated the eQTL observed in the UKBEC dataset between rs7120548 and *MTCH2*, a gene located in the *CELF1* locus (p = 0.0011; Table 5) [22]. In GSE15222, very few genes were present in the cleaned dataset that occur within the GWAS loci for the IGAP SNPs (e.g. *CLU* and *CR1*), making it impossible to independently replicate a subset of eQTLs (S6 Table).

**Table 5:** eQTLs of IGAP GWAS SNPs in GSE15222.

IGAP SNP	IGAP Gene	Analyzed SNP	Gene	P value	β
rs1476679	*ZCWPW1*	rs1476679	*PILRB*	0.0022	0.108811
rs10838725	*CELF1*	rs7120548	*MTCH2*	0.0011	0.07507

Thus, three independent datasets demonstrate that rs1476679 is associated with altered *PILRB* expression in multiple brain regions. Additionally, these datasets provide evidence for a much more complex picture of AD genetic risk than was previously reported in the original IGAP GWAS: (1) the majority of IGAP GWAS SNPs do not significantly affect expression of nearby genes in brain homogenates and (2) eQTLs occur in genes that are near the IGAP SNP but not that have been named as an IGAP gene.

### Identifying the most significant eQTL SNP within IGAP GWAS loci

Because the majority of GWAS top SNPs are in high LD with many SNPs, it is difficult to determine which SNP is the functional variant responsible for modifying LOAD risk. To determine whether other SNPs within the IGAP GWAS loci more significantly contribute to eQTLs, we identified all SNPs within the IGAP GWAS loci with a p-value of 10^−5^ or lower [9]. We then used an unbiased approach to determine which genes are most highly associated with the SNPs within the IGAP GWAS loci (UKBEC; S7 and S8 Tables). Assuming the most stringent cut-off for multiple test correction (p = 10^−6^)[20], we identified SNPs within the *CR1*, *ZCWPW1*, *CLU*, and *PTK2B* loci that produced significant eQTLs (S7 Table).

To determine whether the SNPs that produce the most significant eQTL within each IGAP GWAS locus represent the same signal as the GWAS top SNP or an independent signal, we tested for the association of the IGAP GWAS top SNP with AD risk and then conditioned the analysis based on the most significant eQTL SNP within each locus (S9 Table). Using this approach, in the ADGC subset of the IGAP dataset, we found that for each locus, the most significant eQTL SNP and the IGAP top SNP represented the same signal. Thus, while we identified SNPs within IGAP GWAS loci that produce stronger eQTLs than the IGAP top SNP, these SNPs are likely marking a single risk locus.

### Expression differences in AD brains

To determine whether the named LOAD GWAS genes or genes within the GWAS loci exhibit altered expression in AD brains, we examined gene expression in a study of laser micro-dissected neurons (GSE5281; Table 6). *MTCH2* expression was significantly associated with AD status, where expression levels were lower in AD cases compared with controls (p = 2.2x10^-12^ and 2.9x10^-12^; Table 6; Bonferroni p = 5x10^-4^). *PILRB* expression was also associated with disease status, where *PILBR* expression was lower in AD cases compared with controls (p = 1.2x10^-3^; Table 6). Expression of *GATS*, also within the *ZCWPW1* locus, was associated with AD status in the same direction as *PILRB* (p = 2.1x10^-7^; Table 6).

**Table 6:** Expression of IGAP GWAS loci is associated with disease status in GSE5281.

IGAP Loci	Gene	Probe ID	P values	β
*ZCWPW1*	*ZCWPW1*	223992_x_at	**0.0261**	**-0.3555**
	*ZCWPW1*	220618_s_at	0.8938	0.015
	*PMS2P1*	239699_s_at	0.1083	-0.2038
	*PMS2P1*	214526_x_at	0.7354	-0.0258
	*C7orf51*	1553288_a_at	**0.0156**	**0.2291**
	*C7orf61*	229913_at	**7x10**^**-4**^	**0.4589**
	*C7orf47*	226434_at	0.2368	0.0969
	*MEPCE*	219798_s_at	0.3281	0.0878
	*PILRA*	219788_at	0.2172	0.1819
	*PILRA*	222218_s_at	0.2141	0.1406
	*PILRB*	220954_s_at	**1.2x10**^**-3**^	**-0.5226**
	*PILRB*	225321_s_at	0.0915	0.1579
	*GATS*^#^	227321_at	**2.1x10**^**-7**^	**-0.4098**
*CELF1*	*CELF1*^#^	1555467_a_at	**3.8x10**^**-9**^	**-0.9599**
	*CELF1*	209489_at	0.1711	-0.0928
	*CELF1*	221743_at	**1.5x10**^**-3**^	**0.2706**
	*CELF1*	204113_at	0.4106	-0.1278
	*CELF1*	221742_at	0.317	0.1058
	*CELF1*	235297_at	0.2333	0.2114
	*CELF1*	235865_at	0.8078	0.0408
	*SLC39A13*	225277_at	**3.1x10**^**-3**^	**0.2519**
	*SLC39A13*	1552295_a_at	0.6693	-0.0465
	*PSMC3*^#^	201267_s_at	**4.80x10**^**-6**^	**-0.7725**
	*NDUFS3*^#^	201740_at	**6.6x10**^**-11**^	**-0.8212**
	*MTCH2*^#^	217772_s_at	**2.2x10**^**-12**^	**-0.6621**
	*MTCH2*^#^	222403_at	**2.9x10**^**-12**^	**-0.6235**
	*PTPMT1*^#^	223808_s_at	**2.9x10**^**-7**^	**-0.3872**
	*PTPMT1*^#^	225901_at	**1.2x10**^**-5**^	**-0.7372**
	*PTPMT1*	218570_at	**0.0544**	**-0.1777**
	*AGBL2*	220390_at	0.0813	0.2846
	*FNBP4*	212232_at	**3.1x10**^**-3**^	**-0.4194**
	*FNBP4*	235101_at	**0.0484**	**0.3496**
	*FNBP4*	242472_x_at	**0.0534**	**0.301**
	*FNBP4*	229272_at	0.5129	-0.1087
	*NUP160*^#^	212709_at	**1x10**^**-4**^	**0.4791**
	*NUP160*	214962_s_at	**0.0477**	**-0.3451**
	*NUP160*	214963_at	**0.0587**	**-0.3362**
	*KBTBD4*	218570_at	**0.0544**	**-0.1777**
	*KBTBD4*	218569_s_at	0.0944	-0.25
	*KBTBD4*	223765_s_at	0.3863	0.1439

^**#**^Passed multiple test correction (Bonferroni p = 5x10^-4^)

**Fig 1:**
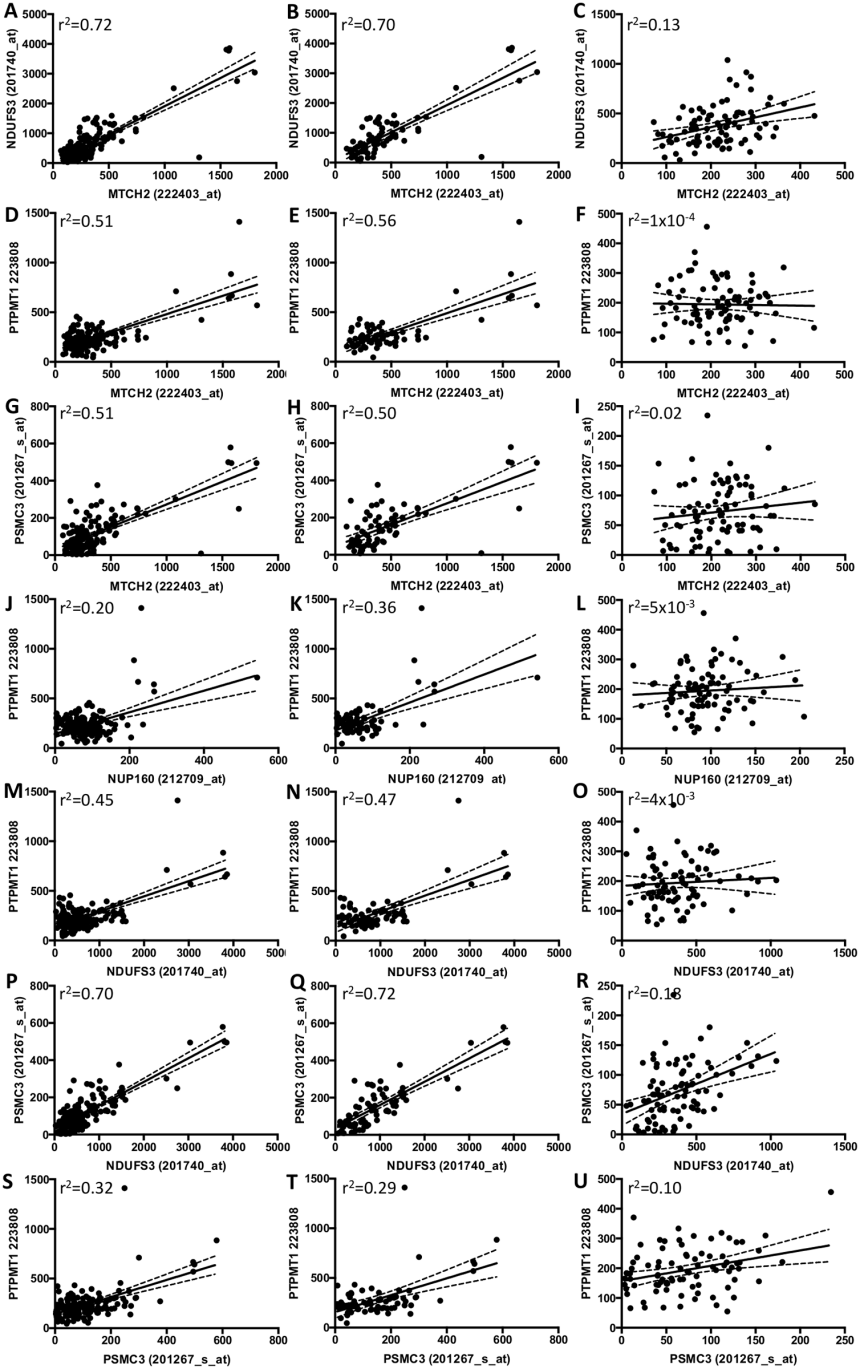
Correlation between expression of genes within the CELF1 locus is lost in AD brains. Expression of *MTCH2*, *NDUFS3*, *PTPMT1*, *PSMC3*, and *NUP160* are highly correlated in laser microdissected neurons. Correlation is lost in AD brains. Gene expression in all brain samples (A, D, G, J, M, P, S). Control only (B, E, H, K, N, Q, T). AD only (C, F, I, L, O, R, U).

Several genes within the GWAS loci were associated with disease status in the neuron-specific expression dataset (GSE5281): *EED*, *POLR2E*, *GPX4*, *SORL1*, *INPP5D*, *MEF2C*, *C7ORF61*, *CELF1*, *PSMC3*, *NDUFS3*, *PTPMT1*, *NUP160*, *C20ORF43*, and *CSTF1* (Table 6; S10 Table). Interestingly, expression of several genes within the *CELF1* GWAS locus were associated with disease status: *CELF1*, *SLC39A13*, *PSMC3*, *PTPMT1*, *NDUFS3*, *MTCH2*, *FNBP4*, and *NUP160*, some of which also produced suggestive evidence of eQTLs (Table 3, S3 and S4 Tables). Expression of *MTCH2*, *NDUFS3*, *PTPMT1*, *PSMC3*, and *NUP160* (but not *CELF1*) were highly correlated in control neurons (Fig 1), and this correlation is lost in AD brains (Fig 1). Interestingly, despite the eQTLs and disease associations with *PILRB* and *GATS* expression, there was no correlation between these genes (S1 Fig). As with our eQTL findings, very few of the genes associated with disease status were the genes originally identified as the gene associated with the IGAP top SNP.

### Cell-type specific expression of genes within the GWAS loci

Evidence from multiple, independent datasets have identified eQTLs between IGAP SNPs and *PILRB* and multiple genes within the *CELF1* locus (including *MTCH2*). We have also observed altered expression levels of *PILRB* and *GATS* within the *ZCWPW1* locus and *MTCH2* and other genes within the *CELF1* locus in AD brains. This could be due to differences in expression within a cell or to differences in the numbers of cells in which these genes are expressed. To determine whether genes within the *CELF1* locus and other IGAP GWAS loci are preferentially expressed in certain cell-types in the brain, we examined a dataset containing RNAseq performed in isolated cell-types in the mouse brain (http://web.stanford.edu/group/barres_lab/brain_rnaseq.html [23]). We found that genes within the *CELF1* locus are most highly expressed in non-neuronal cell-types (S11 Table). This suggests that genes within this region act cooperatively to modify AD risk.

We examined cell-type specific expression of all of the genes within the IGAP GWAS loci (S11 Table). We found that the majority of genes within the IGAP GWAS loci are most highly expressed in microglia (37%): *MEF2C*, *BIN1*, *PICALM*, *CD33*, *CSTF1*, *HLA-DRB1*, *HLA-DQA1*, *HLA-DQB1*, *RIN3*, *INPP5D*, *PILRA*, *SLC39A13*, *CASS4*, and *PTK2B*. To a lesser extent, genes within the IGAP GWAS loci are expressed in endothelial (20%), oligodendrocytes (20%), and astrocytes (17%). Neuronally expressed genes, which have been the central focus of functional studies regarding these and other AD risk genes, only represent 11% of IGAP GWAS loci: *ABCA7*, *MADD*, *CELF1*, and *MEF2C*. These findings provide further evidence of the complex interplay between genotype, expression, and cell-type that mediates AD risk.

## Discussion

Recent studies have identified novel GWAS loci that modulate LOAD risk; however, we still know little of the functional impact of LOAD GWAS SNPs and the role of these genes in AD pathogenesis. In this study, we examined functional effects of IGAP GWAS SNPs by examining eQTLs in several human brain expression cohorts. We found that rs1476679 and rs7120548 are consistently associated with *PILRB* and *MTCH2* expression across multiple cohorts, respectively. Additionally, expression of several genes within the *CELF1* locus, including *MTCH2*, were associated with AD status. From this study, we have generated two important findings: (1) the majority of IGAP GWAS SNPs do not significantly affect expression of nearby genes in human brain homogenates and (2) eQTLs occur in genes that are near the IGAP SNP but that are not named as an AD risk gene.

PILRB is a paired immunoglobin-like type 2 receptor that is involved in regulation of immune response [24]. PILRB contains highly related activating and inhibitory receptors. PILRA is the inhibitory counterpart to PILRB. PILRB, through activation, and PILRA, through inhibition, function cooperatively to control cell signaling via SHP-1, which mediates dephosphorylation of protein tyrosine residues. PILRA and PILRB are mainly expressed by cells of the myeloid lineage [24]. PILRB associates with DAP12, a signaling adaptor protein that is cleaved by γ-secretase and associates with TREM2, another AD risk gene [25–28]. PILRB also contains a sialic acid binding domain, similar to the one described for CD33 [11, 14, 29]. Rs1476679 produced an eQTL with *PILRB* transcripts in human brain homogenates as well as in monocytes (Table 1)[30], suggesting that this AD risk SNP may influence *PILRB* expression in microglia in the brain.

One hypothesis based on our observation that multiple genes within the *CELF1* loci have eQTLs or are associated with AD status is that there is a key regulator within this region that is influencing the expression of many genes. MTCH2 is a mitochondrial carrier protein that induces mitochondrial depolarization [31]. MTCH2 associates with truncated BID to activate apoptosis [31]. MTCH2 interacts with presenilin 1 [32, 33]. A second mitochondrial protein that displayed some eQTL evidence and association with disease status, *NDUFS3*, also occurs within the *CELF1* locus. NDUFS3 is a component of the NADH-ubiquinone oxidoreductase (Complex 1). NDUFS3 occurs in KEGG pathways for AD, Parkinson’s disease, and Huntington’s disease (KO05010, KO05012, KO05016). The third gene within this locus, *NUP160*, with some evidence of an eQTL and altered expression in AD brains, is a key component of the nuclear pore complex, which mediates nucleoplasmic transport. NUP160 has an extremely long half-life and is thus susceptible to oxidative and age-related damage. Age-related defects in NUP160 and the nuclear pore complex has been proposed to contribute to abnormal protein trafficking, and in turn to neurodegenerative diseases [34, 35]. PTPMT1 is a lipid phosphatase that dephosphorylates mitochondria proteins, which in turn regulates mitochondrial membrane integrity. PSMC3 encodes the 26S proteasomal subunit, which plays a critical role in ATP-dependent degradation of ubiquitinated proteins. *MTCH2*, *NUP160*, *NDUFS3*, *PTPMT1*, and *PSMC3* expression are highly correlated in human brains and this correlation is lost in AD brains.

Our cell-type specific expression studies illustrate that the majority of the genes expressed within the IGAP GWAS loci are most highly expressed in microglia. These findings illustrate the important role of immune response and clearance in LOAD pathogenesis. This has been further supported in recent studies demonstrating that genetic variants linked with neurodegeneration are more likely to affect gene regulation in monocytes than in T cells [36].

One caveat to the study is that not all of the genes present within all of the IGAP loci were present in the cleaned expression dataset for GSE15745. Thus, we cannot exclude the possibility that other genes within the loci also have significant eQTLs with the IGAP SNPs. Most of these eQTL studies are also based on RNA extracted from brain homogenates, thus eQTLs in cells that represent a minority of cells within that tissue homogenate may not be detectable using this approach. It also remains possible that GWAS SNPs drive changes at the protein level or drive transient changes in human brains. However, our findings of several strong associations with IGAP SNPs and expression of genes that were not named as AD risk genes emphasizes that the IGAP SNPs with putative functional effects may act on genes within the GWAS loci rather than the genes immediately under the most significant IGAP SNP.

## Methods

### Publically available expression datasets

#### GSE15745

The GSE15745 dataset was obtained from control brains [21]. Brains from 150 neurologically normal individuals of European descent were obtained from the Department of Neuropathology, Johns Hopkins University, Baltimore and from the Miami Brain Bank. Brain tissue was collected from the cerebellum, frontal cortex, pons and the temporal cortex. The samples were 31.3% female with a mean age of 45.8 years (range 15–101) and an average PMI of 14.3 hours. SNP genotyping was performed on DNA extracted from cerebellar tissue for each subject using Infinium HumanHap550 version 3 BeadChips. RNA expression was measured using HumanRef-8 Expression BeadChips (Illumina). To analyze RNA expression residual values were used that were log transformed and incorporated gender, age, and PMI as covariates [21].

#### GSE15222

The GSE15222 dataset was used to examine eQTLs [37]. Neuropathologically confirmed AD (n = 176) or normal controls (n = 188) of self-identified individuals of European descent, were obtained from 20 National Alzheimer's Coordinating Center (NACC) brain banks and from the Miami Brain Bank. The 188 control brains came from one of three brain regions: 21% frontal cortex, 73% temporal cortex and 2% parietal cortex. The samples were 45% female with a mean age of 81 years (range 65–100) and an average post mortem interval (PMI) of 10 hours. The 176 LOAD brains were composed of 18% frontal cortex, 60% temporal cortex and 10% parietal cortex. The samples were 50% female with a mean age of 84 years (range 68–102) and an average PMI of 9 hours. An Affymetrix 500K chip was used to obtain genotype data, and an Illumina ref-seq 8 chip was used to obtain RNA expression data. To analyze RNA expression, residual values were used that were log transformed and then gender, *APOE* genotype, age, hybridization date, site, and PMI were included as covariates.

#### GSE5281

The GSE5281 dataset was obtained from laser microdissected neurons from AD and control brains [38]. Brain samples from 47 individuals of European descent that were collected from Washington University, Duke University, and Sun Health Research Institute were included in the study. Samples were clinically and neuropathologically confirmed AD or controls. The 33 AD samples were 54.5% female with a mean age of 79.9 years (range 73–86.8) and an average PMI of 2.5 hours. The 14 control brains were 28.6% female with a mean age of 79.8 years (range 70.1–88.9). All samples were obtained from the entorhinal cortex, hippocampus, medial temporal gyrus, posterior cingulate, superior frontal gyrus, and primary visual cortex. RNA expression was measured using an Affymetrix GeneChip for gene expression. To analyze RNA expression, the log transformed expression values were analyzed with brain region, age, and gender as covariates.

#### UKBEC

The UKBEC (www.braineac.org) dataset is composed of brains from 134 neuropathologically normal controls [20]. Ten brain regions were extracted for each brain: occipital cortex (OCTX), frontal cortex (FCTX), temporal cortex (TCTX), hippocampus (HIPP), intralocular white matter (WHMT), cerebellar cortex (CRBL), thalamus (THAL), putamen (PUTM), substantia nigra (SNIG), and medulla (MEDU). RNA expression was measured using an Affmetrix Exon 1.0 ST array. Genotyping was performed on the Illumina Infinium Omni1-Quad BeadChip.

### IGAP LOAD GWAS

International Genomics of Alzheimer's Project (IGAP) is a large two-stage study based upon genome-wide association studies (GWAS) on individuals of European ancestry. In stage 1, IGAP used genotyped and imputed data on 7,055,881 single nucleotide polymorphisms (SNPs) to meta-analyze four previously-published GWAS datasets consisting of 17,008 Alzheimer's disease cases and 37,154 controls (The European Alzheimer's disease Initiative–EADI the Alzheimer Disease Genetics Consortium–ADGC The Cohorts for Heart and Aging Research in Genomic Epidemiology consortium–CHARGE The Genetic and Environmental Risk in AD consortium–GERAD). In stage 2, 11,632 SNPs were genotyped and tested for association in an independent set of 8,572 Alzheimer's disease cases and 11,312 controls. Finally, a meta-analysis was performed combining results from stages 1 & 2.

### ADGC

The ADGC case-control database was previously described [8]. The 15 datasets with imputed data were analyzed (1,000 Genomes Project Phase 1 March 2012 v3).

### Statistical analysis

Relative gene expression values were log transformed to achieve a normal distribution. To identify covariates that influence the expression of each gene, a stepwise discriminant analysis was performed using CDR, age, gender, disease status, PMI (post mortem interval), RIN (RNA integrity number), and *APOE* genotype. After applying the appropriate covariates to the model, analysis of covariance (ANCOVA) was used to test for association between genotypes and gene expression. SNPs were tested using an additive model. All analyses were performed using statistical analysis software (SAS). Conditional analyses were performed by adjusting for the most significant eQTL SNP within each IGAP GWAS locus to determine whether the eQTL SNP represented an independent association. Additional covariates included in the analyses were age, gender, principal components 1–3, and site.

## Supporting Information

S1 Fig No correlation is observed between PILRA, PILRB, and GATS in human brains. Expression of PILRA, PILRB, and GATS were plotted in laser microdissected neurons.
(PDF)

S1 Table RegulomeDB Scores. (XLSX)

S2 Table eQTLs of IGAP GWAS SNPs in control brains (UKBEC). (XLSX)

S3 Table Most Significant eQTLs for top IGAP SNPs. (XLSX)

S4 Table eQTLs of genes within IGAP GWAS loci in control brains (GSE15745). (XLSX)

S5 Table Linkage Disequilibrium of SNPs Analyzed in GSE15222 and GSE15745. (XLSX)

S6 Table eQTLs of IGAP GWAS SNPs in GSE15222. (XLSX)

S7 Table Most significant eQTLs in IGAP loci in control brains (UKBEC). (XLSX)

S8 Table Top eQTLs for SNPs within IGAP Loci in Control Brains (UKBEC). (XLSX)

S9 Table Conditional analysis of SNPs producing the most significant eQTLs in control brains. (XLSX)

S10 Table Expression of IGAP GWAS loci is associated with disease status in GSE5281. (XLSX)

S11 Table Cell-type specific expression of genes within the IGAP GWAS loci. (XLSX)
