# Supplementary figures and images for: Alzheimer’s Disease Risk Polymorphisms Regulate Gene Expression in the ZCWPW1 and the CELF1 Loci
Source: PLoS One. 2016 Feb 26;11(2):e0148717. doi: 10.1371/journal.pone.0148717 (PMC4769299; doi:10.1371/journal.pone.0148717)

S1 Fig

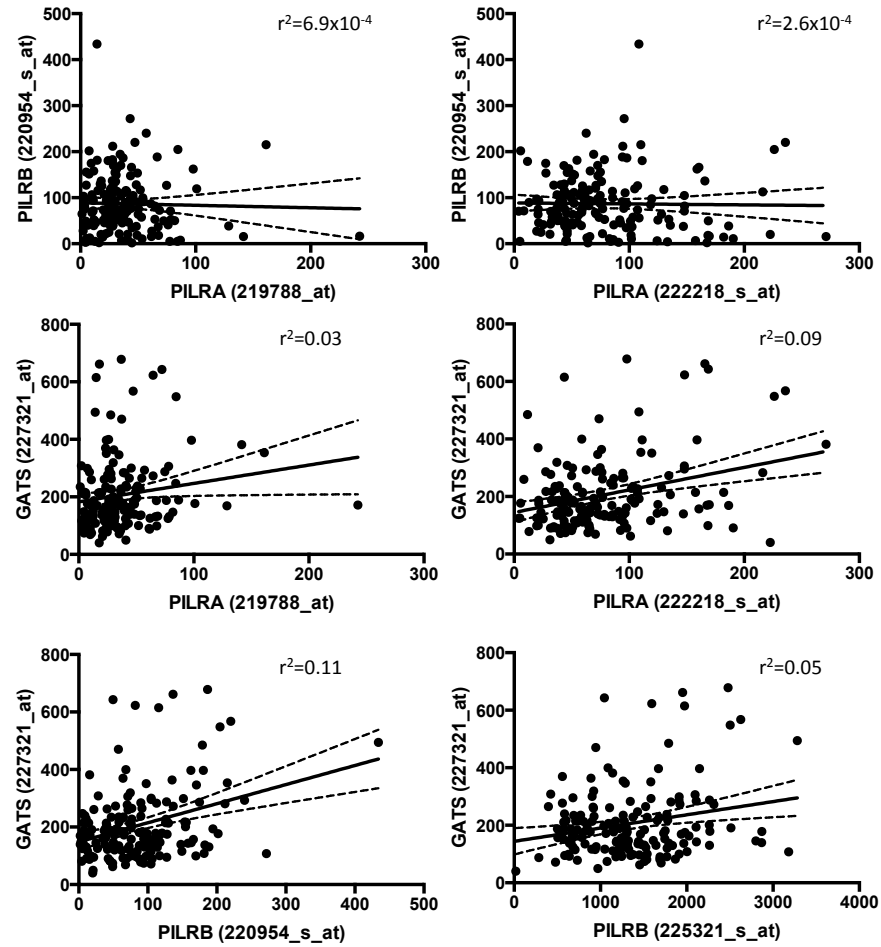

Supplement: S1 Fig — Expression of PILRA, PILRB, and GATS were plotted in laser microdissected neurons. (PDF) [file pone.0148717.s001.pdf]
